# Prospective associations between strengths of moral character and health: longitudinal evidence from survey and insurance claims data

**DOI:** 10.1007/s00127-022-02344-5

**Published:** 2022-08-02

**Authors:** Dorota Weziak-Bialowolska, Matthew T. Lee, Piotr Bialowolski, Ying Chen, Tyler J. VanderWeele, Eileen McNeely

**Affiliations:** 1 Sustainability and Health Initiative (SHINE), Department of Environmental Health, Harvard T. H. Chan School of Public Health, Boston, MA USA; 2 Human Flourishing Program, Institute for Quantitative Social Science, Harvard University, Cambridge, MA USA; 3 Centre for Evaluation and Analysis of Public Policies, Faculty of Philosophy, Jagiellonian University, Cracow, Poland; 4 Department of Economics, Kozminski University, Warsaw, Poland; 5 Department of Epidemiology, Harvard T. H. Chan School of Public Health, Boston, MA USA; 6 Institute for Studies of Religion, Baylor University, Waco, TX, USA

**Keywords:** Strengths of moral character, Use of character strengths, Depression, Anxiety, Mental health, Physical health

## Abstract

**Purpose:**

Excellent character, reflected in adherence to high standards of moral behavior, has been argued to contribute to well-being. The study goes beyond this claim and provides insights into the role of strengths of moral character (SMC) for physical and mental health.

**Methods:**

This study used longitudinal observational data merged with medical insurance claims data collected from 1209 working adults of a large services organization in the US. Self-reported physical and mental health as well as diagnostic information on depression, anxiety, and cardiovascular disease were used as outcomes. The prospective associations between SMC (7 indicators and a composite measure) and physical and mental health outcomes were examined using lagged linear and logistic regression models. A series of sensitivity analyses provided evidence for the robustness of results.

**Results:**

The results suggest that persons who live their life according to high moral standards have substantially lower odds of depression (by 21–51%). The results were also indicative of positive associations between SMC and self-reports of mental health (β = 0.048–0.118) and physical health (β = 0.048–0.096). Weaker indications were found for a protective role of SMC in mitigating anxiety (OR = 0.797 for the indicator of delayed gratification) and cardiovascular disease (OR = 0.389 for the indicator of use of SMC for helping others).

**Conclusions:**

SMC may be considered relevant for population mental health and physical health. Public health policies promoting SMC are likely to receive positive reception from the general public because character is both malleable and aligned with the nearly universal human desire to become a better person.

**Supplementary Information:**

The online version contains supplementary material available at 10.1007/s00127-022-02344-5.

## Introduction

Following Aristotle [1], some scholars argue that an excellent character, reflected in high standards of moral behavior, as well as an orientation to promote good and engage in good deeds even in adverse circumstances, may contribute to complete well-being [2–5]. Empirical evidence has already corroborated a positive association between strengths of the character and self-assessed physical health and mental health [6, 7], physical fitness [8], life satisfaction [9], subjective and psychological well-being [7, 10–13], and decreased depression [9, 14, 15]. The associations between character strengths and specific physical diseases have also been examined. For example, there is some experimental evidence that application of character strengths can be helpful in improving pain self-efficacy and capacity to function with pain [16]. A rigorous review of clinical studies on character strengths-based interventions for patients with chronic illnesses revealed that these interventions boosted self-esteem and self-efficacy and reduced depression [17]. Character strengths were found to be positively associated with improved quality of life among people with multiple sclerosis [18] and among patients with an acute coronary syndrome [19]. They were also reported as an important factor in moderating the relationship between COVID-19 stress and well-being among individuals with chronic health conditions and disabilities [20]. Finally, one specific character strength—the character strength of honesty and integrity—was also found to be prospectively associated with lower risk of lung disease, lower limitations in mobility and less difficulty in instrumental activities of daily living among middle-age and older adults [15].

Although previous studies have substantially advanced our understanding of the potential role of character strengths for human flourishing, they are subject to certain limitations. First, previous studies focused on the impacts of character strengths on subjective well-being and self-reported health outcomes, and thus provided limited evidence on their association with objectively measured health conditions, though theoretical considerations are supportive of positive impacts [21–23]. Second, experimental evidence on the impact of character strengths on mental health is ambiguous. While some authors reported contributions to decreased depressive symptoms [14, 17], others found no such impact [24, 25]. Third, although some recent longitudinal evidence provides reasonable support for the prospective associations between character strengths and well-being and/or health [6, 26], numerous observational studies concerning character strengths often relied on cross-sectional datasets and provided findings of a correlational nature, which have been already shown to overestimate the magnitude of the actual relationship [27].

In order to overcome these limitations, at least partially, this study examines temporal associations between adherence to high standards of moral behavior and both mental and physical health outcomes using panel survey data merged with diagnostic information derived from the insurance claims data.

We hypothesize that adherence to high standards of moral behavior is favorably associated with subsequent lower risk of disease (i.e., diagnosed depression, diagnosed anxiety and diagnosed cardiovascular disease), as well as with increased self-reports of physical and mental health even after adjusting for a wide range of potential confounders.

## Materials and methods

### Data

We used two waves of survey data merged with the diagnostic information on medical conditions included in the medical insurance claims data. Specifically, a group of randomly sampled working adults of a large, national service organization based in the United States provided survey data at two occasions. In the first wave, conducted in June 2018, 2370 individuals provided responses. First wave participants were subsequently invited to the second wave of the study and provided responses in July 2019. The number of participants in both waves amounted to 1,209, which yielded the retention rate of 51.2%. Among participants, females accounted for 84.5% vs. 74.5% for the entire population, which reflected the feminization rate in the organization. Mean age of participants was 43.5 years in the sample compared to 45.6 in the population.

The survey was designed to comprehensively assess well-being and work conditions among employees. It was administered online, which allowed participants to choose a secure and anonymous space to participate in the study. Eligibility criteria for participation included age (i.e., at least 18 years of age) and employment status (i.e., all current employees were considered). Participation was voluntary, confidential, and conditional on the informed written consent that was collected from each participant. Harvard Longwood Campus Institutional Review Board reviewed and approved all protocols for the study. More information about the study and sample is presented elsewhere [6, 28, 29].For respondents who participated in wave 1 (*T* = 1) and wave 2 (*T* = 2), we merged their survey records with their medical insurance claims data (*T* = 0, *T* = 1, and *T* = 2) that were provided by the employer. Next to a number of financial measures such as allowed amounts for medical services and pharmacy products, medical insurance data included data on medication prescribed and diagnostic information on medical conditions, which were of interest in this study. Diagnostic information followed the International Classification of Diseases (ICD-10) [30]. It has been also demonstrated to be highly consistent with medical records and useful in epidemiological studies [31, 32]. Merged survey and medical insurance data have been already found useful in other research addressing well-being and health [28]. Table 1 (adapted from [28]) presents the descriptive statistics at baseline (*T* = 1). Data are available on reasonable request.

**Table 1:** Participant characteristics at study baseline (*T* = 0, survey data 2018–2019 merged with health insurance data 2017–2019, United States, *N* = 1209)

Baseline characteristic	*n*	Statistic
Gender, %		
Women	1021	84.45
Men	188	15.55
Age—mean (SD)		43.52 (10.4)
Age, %		
Below 30	143	11.83
31–40	362	29.94
41–50	350	28.95
Above 50	354	29.28
Race, %		
White	898	74.28
Black or African American	147	12.16
Hispanic/Latino	81	6.70
Asian	61	5.05
Other	22	1.81
Marital status (married), %		
Married	744	62.47
Single, never married	193	16.20
Divorced	120	10.08
Non-married partner	103	8.65
Widowed	16	1.34
Separated	15	1.26
Education, %		
High school	93	7.78
Some college but no degree	270	22.58
Associate degree	167	13.96
Bachelor’s degree	418	34.95
Graduate school or higher	248	20.74
Having children under the age of 18 currently living in the household, %		
Yes	574	48.11
No	619	51.89
Being a primary caregiver for a parent or an elderly currently living in the household, %		
Yes	325	27.17
No	871	72.83
Home ownership, % of yes		
Yes	330	72.36
No	864	27.64
Salary (USD)—mean (SD)	1209	73,117 (34,259)
Voting in the previous elections, % of yes		
Yes	980	81.94
No	216	18.06
Religious service attendance, %		
At least once/week	245	20.48
Less than once/week	615	51.42
Never	336	28.09
Spiritual practicing, %		
At least once/week	633	61.15
Less than once/week	466	30.66
Never	98	8.19
Volunteering, %		
At least once/week	757	9.72
Less than once/week	116	63.40
Never	321	26.88
Participating in community groups, %		
At least once/week	121	18.49
Less than once/week	593	49.62
Never	381	31.88
Participating in a medical plan, %		
Yes	1042	86.19
No	167	13.81

Adapted from “The role of financial conditions for physical and mental health. Evidence from a longitudinal survey and insurance claims data” by Bialowolski P, Weziak-Bialowolska D, Lee MT, Chen Y, VanderWeele TJ, McNeely E. (2021) *Social Science & Medicine*; 281:114,041. (https://doi.org/10.1016/j.socscimed.2021.114041). CC BY-NC-ND

### Measures

#### Mental health outcomes

We examined one self-reported mental health outcome from the Well-Being Assessment (WBA)^1^ [29, 33] and the Flourishing Index [4, 34] [‘*In general, how would you rate your mental health?*’ (0 = poor and 10 = excellent)] and two mental health outcomes captured in health insurance claims data, that is: (1) diagnosis of depression (yes vs. no) and/or (2) diagnosis of anxiety (yes vs. no).

#### Physical health outcomes

We examined one self-reported physical health outcome from the WBA [29, 33] and the Flourishing Index [4, 34] [‘*In general, how would you rate your physical health?*’ (0 = poor and 10 = excellent)] and one diagnostic information on medical conditions outcomes derived from the participants’ medical insurance claims data, i.e., a diagnosed cardiovascular disease (yes vs. no).

#### Strengths of moral character

To measure adherence to high standards of moral behavior, a subscale of the WBA, related to strengths of moral character (SMC), was used*.* The SMC-WBA instrument was developed based on the concept of human flourishing or complete well-being [4, 35]. The SMC domain, which is of interest in this study, was conceptualized according to a long-standing religious and philosophical tradition, partially adopted by positive psychology in recent years, positing that in order to attain complete well-being, an excellent character and acting in accordance with the virtue, are essential [1, 13, 14, 36, 37]. Consequently, this domain was defined as adherence to high standards of moral behavior reflected in an ability to focus, to maintain consistent thoughts, and to act in a way that contributes to the good of oneself and others [33]. High score in SMC-WBA indicates a self-assessed “strength” in moral character.

SMC-WBA is related to the concept of ‘character strengths’ in general [37] and to one popular measure of character strengths specifically—the VIA Survey of Character Strengths [36]. We refer to our assessment as a ‘measure of strengths of moral character’ to highlight its moral component and to distinguish it from the VIA measure of character strengths. In the Supplementary Information, we present details on similarities and dissimilarities between our measure and the VIA Survey of Character Strengths [36].

Five aspects of adherence to high standards of moral behavior were examined with seven statements from the SMC-WBA [29, 33]:

1. moral compass (*‘I always know the right thing to do’*),
2. orientation to promote good (‘*I am willing to face difficulties in order to do what is right’*, *‘I give up personal pleasures whenever it is possible to do some good instead’*, and *‘I always act to promote good in all circumstances, even in difficult and challenging situations’),*
3. use of strengths (*‘I get to use my strengths to help others’),*
4. kindness (*‘I always treat everyone with kindness, fairness and respect’*) and
5. delayed gratification (*‘I am always able to give up some happiness now for greater happiness later’)*.

Respondents could choose an answer on a 0 = ‘not true of me’ to 10 = ‘completely true of me’ scale. The seven items of the SMC-WBA were moderately correlated (*r* = 0.36–0.61; correlations between main study variables are presented in Table A1 in the Supplementary Information). In addition, SMC-WBA (an aggregate of seven items from the SMC-WBA) was also used as an exposure. This scale was validated and showed satisfactory psychometric properties in terms of reliability (alpha = 0.88), test–retest correlation (*r* = 0.67), and convergent/discriminant validity in relation to stability over time (*r* = 0.75), as well as a good fit to the data (confirmatory factor analysis: CFI = 0.962, TLI = 0.943, RMSEA = 0.069) that were invariant over time, gender, age, education, and marital status [a complete psychometric evaluation can be found in 33].

#### Control variables

A rich set of control variables was used. Specifically, we controlled for demographic characteristics [gender (male, female), age group (≤ 30, 31–40, 41–50, > 50), race (White, Black/African American, Hispanic/Latino, Asian, other), educational attainment (high school, some college, associate degree, bachelor’s degree, graduate degree), marital status (married vs. not married), having children at home (yes vs. no), taking care of an elderly (yes vs. no)], wealth [owning a house (yes vs. no)], and income [salary (the mid-point salary bands were provided by the employer)]. These variables are classified as social determinants of health and, as shown by previous research [38, 39], have a substantial impact on people’s health, well-being and quality of life. In addition, we controlled for social participation and civic engagement. The variables comprised: (1) voting in the last elections (yes vs. no/not registered voter), (2) religious service attendance (at least once a week, less than once a week, never), (3) spiritual practices (at least once a week, less than once a week, never), (4) volunteering (at least once a week, less than once a week, never), and (5) community work (at least once a week, less than once a week, never). In prior studies, these factors were found to play a predictive role for health and well-being [40–44,  77].

Next, since the impact of work on health has long been recognized in theory [45] and empirical research [46–50, 78], we controlled for work characteristics. We included selected indicators of work resources, work demands and work autonomy: number of work hours, supervisor support [*‘My supervisor supports me’* (0–10)], job control [*‘I have a lot of freedom to decide how to do my job’* (0–10)], job demand [*‘I have too much to do at work to do a good job’* (0–10)], job fit [*‘At work, I am able to do what I am good* at’ (0–10)] and job meaning [*‘I find my work meaningful’* (0–10)] [51, 52]. These variables were controlled for in the first wave (*T* = 1). In addition, in each regression, the control was made for an outcome and additionally for the number of diagnosed health conditions prior to exposure to further reduce possibility of reverse causality.

### Statistical analysis

This study applied an outcome-wide analytic approach [53] and used longitudinal observational data merged with medical insurance claims data. The logistic (for dichotomous outcomes) and linear (for continuous outcomes) regression analysis was applied. All continuous outcomes were standardized (i.e., mean = 0, standard deviation = 1), to report the effect estimates in terms of standard deviations of the outcome variables (i.e., standardized effect sizes). For dichotomous outcomes, we presented odds ratios.

A set of 40 regression models was used to regress each of the five outcomes on each of the eight exposures (i.e., SMC-WBA and its seven items) separately. In particular, the association between a character strength exposure *j* and a health outcome *k* for continuous outcomes was modeled as follows:

1 $${HO}_{i,k}\left(T=2\right)={{\alpha }_{0}+{{\alpha }_{1}{SMC}_{j,i}\left(T=1\right)+\alpha }_{2}X}_{i}\left(T=1\right)+{\alpha }_{3}{HO}_{i,k}\left(T=1\right)+{\eta }_{k,j,i},$$

and for dichotomous outcomes as follows:

2 $${prob[HO}_{i,k}(T=2)=1]=\frac{1}{1+{\mathrm{e}}^{-\left({{\alpha }_{0}+{{\alpha }_{1}{\mathit{SMC}}_{j,i}\left(T=1\right)+\alpha }_{2}X}_{i}\left(T=1\right)+{{\alpha }_{3}{\mathit{HO}}_{i,k}\left(T=0\right)\eta }_{k,j,i}\right)}},$$

where *i* = 1,…,*N*; *k* = 1,…,5*; j* = 1,…,8.

Subscript *i* represents an individual, the variable *HO* indicates one out of five (*k* = 1,…,5) health outcomes, *SMC* is one out of eight exposures (*j* = 1,…,8). *X* is a vector of control variables. *α*_*1*_ reflects an association between SMC exposure and a subsequent health outcome. *α*_*2*_ shows the association between control variables and the health outcome. *α*_*3*_ shows the association between the health outcome *k* at *T* = 2 and *T* = 1 for self-reported health outcomes and at *T* = 2 and *T* = 0 for medical condition outcomes. *η*_*k,j*_*,*_*i*_ is a disturbance term.

All missing exposure, covariate, and outcome variables were imputed using chained Eqs. (20 datasets were generated) [54, 55]. Data were arranged in a wide format as suggested by Allison [56] and all outcome, exposure and control variables were used in the procedure. Consequently, the multiple imputation estimates pooled using the Rubin’s formula [57] are presented. Bonferroni correction was used to correct for multiple testing.

Robustness of the results was examined through a series of robustness analyses. First, for three regressions of diagnosed conditions (i.e., depression, anxiety, and a cardiovascular disease, derived from the medical insurance diagnostic information), supplementary analyses were conducted on a limited sample of those who did not suffer from the health outcome under examination prior to exposure (as opposed to the primary analysis of the entire sample controlling for the outcomes prior to exposure; Table A2 in the Supplementary Information). Second, two additional sets of controls were added to the primary set of analyses: (1) an alternative specification of the overall 2018 well-being index; it was calculated excluding the character strength specific domain in 2018 (Table A3 in the Supplementary Information), (2) all five well-being domain-specific scores in 2018 (the character strength specific domain score was excluded; Table A4 in the Supplementary Information). Third, we reanalyzed the primary sets of models using the complete-case analysis (Table A5 in the Supplementary Information) to examine robustness of the results to the missing data patterns. Fourth, because our choice of using a broad set of controls might have contributed to overfitting the models, we rerun them excluding particular sets of confounders (Table A6 in the Supplementary Information). In model 1, we controlled only for social determinants of health (i.e., demographic characteristics, wealth, and income). In model 2, compared to model 1, we added social participation and civic engagement (i.e., we controlled for demographic characteristics, wealth, income, social participation, and civic engagement). To decrease the risk of reverse causation, both model 1 and model 2 also controlled for the prior outcomes and the history of disease. Fifth, we rerun the primary models using all items of SMC-WBA simultaneously to examine the overall effect of the co-occurrence of different aspects of SMC (Table A7 in the Supplementary Information). Finally, the sensitivity measures—*E* values—were calculated to assess the robustness of the observed associations to unmeasured confounding [58, 59].

Analyses were performed using Stata/SE 17.0 for Mac.

## Results

### Characteristics of the study participants in terms of strengths of character and health

In 2019, participants on average reported higher scores in terms of all measured aspects of their strengths of moral character (in each case *p* value < 0.001; one-sided *t* tests for paired observations) and self-assessed physical health (*p* < 0.001) in comparison to 2018 (Table 2, [28]). No improvement in self-reported mental health was noted (*p* = 0.094) but the prevalence of depression increased between 2017 and 2019. No significant changes in the prevalence of cardiovascular disease or of anxiety were found.

**Table 2:** Evolution of strengths of moral character and health outcomes (survey data 2018–2019 merged with health insurance data 2017–2019, United States, *N* = 1209)

Characteristic	2017	2018	2019	*p* value and *t* statistic for one-sided *t* test and effect size for paired observations	
				2017–2018	2018–2019
Strengths of moral character					
Moral compass					
I always know the right thing to do (0–10); mean (SD)	–	7.44 (1.73)	7.87 (1.57)	–	< 0.001; 8.65; 0.25
Orientation to promote good					
I am willing to face difficulties in order to do what is right (0–10); mean (SD)	–	8.34 (1.35)	8.47 (1.31)	–	< 0.001; 3.29; 0.10
I give up personal pleasures whenever it is possible to do some good instead (0–10); mean (SD)	–	7.33 (1.82)	7.75 (1.61)	–	< 0.001; 9.13; 0.26
I always act to promote good in all circumstances, even in difficult and challenging situations	–	8.06 (1.62)	8.34 (1.47)	–	< 0.001; 6.84; 0.20
Use of strengths					
I get to use my strengths to help others (0–10); mean (SD)	–	7.96 (1.69)	8.13 (1.53)	–	< 0.001; 3.74; 0.11
Kindness					
I always treat everyone with kindness, fairness and respect		8.57 (1.32)	8.71 (1.32)	–	< 0.001; 4.22; 0.12
Delayed gratification					
I am always able to give up some happiness now for greater happiness later	–	7.77 (1.60)	8.08 (1.50)	–	< 0.001; 6.63; 0.19
Strengths of moral character scale (SMC-WBA)	–	7.92 (1.17)	8.19 (1.16)	–	< 0.001; 9.42; 0.28
Health outcomes					
Self-reported					
Mental health (0–10); mean (SD)	–	7.59 (1.87)	7.65 (1.81)	–	0.094; 1.32; 0.04
Physical health (0–10); mean (SD)	–	5.88 (1.76)	7.25 (1.73)	–	< 0.001; 31.1; 0.90
Diagnostic information on medical conditions from the health insurance data					
Depression, %	9.59	10.42	12.65	0.070; 1.48; 0.04	< 0.001; 3.36; 0.10
Anxiety, %	12.66	12.16	13.40	0.760; − 0.71, − 0.02	0.062; 1.54, 0.04
Cardiovascular disease, %	1.82	2.15	2.31	0.124; 1.15; 0.03	0.297; 0.53; 0.02

“––” stands for outcome not measured; degrees of freedom vary between 1,185 and 1,208 depending on the variable. Adapted from “The role of financial conditions for physical and mental health. Evidence from a longitudinal survey and insurance claims data” by Bialowolski P, Weziak-Bialowolska D, Lee MT, Chen Y, VanderWeele TJ, McNeely E. (2021). Social Science & Medicine; 281:114,041. (https://doi.org/10.1016/j.socscimed.2021.114041). CC BY-NC-ND

### Strengths of moral character and mental health

Moral compass was found to be significantly associated with subsequent reduced odds of depression (Table 3). After adjusting for covariates and previous history of depression, each standard deviation increase in moral compass was associated with a 31% reduced odds of depression (OR = 0.694, 95% CI 0.554, 0.869) over a 1-year follow-up period. Orientation to promote good was positively associated with subsequent self-assessed mental health and inversely with subsequent onset of depression. With respect to diagnosed depression, each standard deviation increase in orientation to promote good was associated with a 30% reduced odds of depression in the case of being willing to face difficulties in order to do what is right (OR = 0.703, 95% CI 0.512, 0.837), a 37% reduced odds for giving up personal pleasures whenever it is possible to do some good instead (OR = 0.626, 95% CI 0.492, 0.798), and a 26% reduced odds for always acting to promote good in all circumstances, even in difficult and challenging situations (OR = 0.735, 95% CI 0.584, 0.925), over a 1-year follow-up period and after adjusting for covariates and previous history of depression. Self-assessed mental health was associated with prior orientation to promote good with the effect sizes ranging from 0.048 (for ‘*I give up personal pleasures whenever it is possible to do some good instead*’) to 0.083 (for ‘*I always act to promote good in all circumstances, even in difficult and challenging situations*’).

**Table 3:** Associations between strengths of moral character and subsequent health (survey data 2018–2019 merged with health insurance data 2017–2019, United States, *N* = 1209)

Strengths of moral character (0–10)	Mental health outcome			Physical health outcome	
	Self-reported mental health	Anxiety	Depression	Self-reported physical health	Cardiovascular disease
	β^a^ 95% CI	OR 95% CI	OR 95% CI	β^a^ 95% CI	OR 95% CI
Moral compass					
I always know the right thing to do	0.019 (− 0.028, 0.067)	0.881 (0.719, 1.077)	0.694*** (0.554, 0.869)	0.019 (− 0.028, 0.067)	0.976 (0.484, 1.967)
Orientation to promote good					
I am willing to face difficulties in order to do what is right	0.062* (0.014, 0.109)	0.866 (0.706, 1.063)	0.703** (0.512, 0.837)	0.039 (− 0.009, 0.087)	0.795 (0.399, 1.582)
I give up personal pleasures whenever it is possible to do some good instead	0.048** (0.002, 0.095)	0.930 (0.749, 1.155)	0.626*** (0.492, 0.798)	0.027 (− 0.021, 0.075)	0.957 (0.482, 1.900
I always act to promote good in all circumstances, even in difficult and challenging situations	0.083*** (0.034, 0.132)	0.927 (0.752, 1.142)	0.735** (0.584, 0.925)	0.076** (0.028, 0.125)	1.266 (0.562, 2.849)
Use of strengths					
I get to use my strengths to help others	0.061** (0.010, 0.113)	0.986 (0.786, 1.238)	0.619*** (0.481, 0.797)	0.084** (0.021, 0.136)	0.389* (0.186, 0.811)
Kindness					
I always treat everyone with kindness, fairness and respect	0.059* (0.013, 0.104)	1.002 (0.809, 1.240)	0.793* (0.633, 0.993)	0.034 (− 0.012, 0.080)	0.940 (0.444, 1.991)
Delayed gratification					
I am always able to give up some happiness now for greater happiness later	0.046 (− 0.001, 0.094)	0.797* (0.650, 0.976)	0.721** (0.573, 0.908)	0.048* (0.000, 0.095)	1.157 (0.545, 2.456)
Strengths of moral character scale (SMC-WBA)	0.118*** (0.048, 0.188)	0.820 (0.609, 1.104)	0.487*** (0.350, 0.678)	0.096** (0.027, 0.165)	0.737 (0.270, 2.010)

A set of regression models was used to regress each outcome on each character strength exposure separately, to estimate odds ratio (OR) for binary outcomes or β (for continuous outcomes). Each analysis was controlled for demographics (gender, age, race, education, marital status, having children at home, taking care of an elderly), wealth and income (home ownership and salary), lifestyle (voting in the last elections, religious service attendance, spiritual practices, volunteering, community work) and work characteristics (number of work hours, supervisor support, job control, job demand and job meaning). These variables were controlled for in the first wave (in the same wave as the exposure), since only two waves of survey data were available. In addition, in each regression, an outcome prior to exposure as well as the number of diagnosed health conditions (ranging from 0 to 37 possible diagnosed health conditions) prior to exposure were applied as controls

^***^p < 0.001, **p < 0.01, *p < 0.05; the *p* value cutoff for Bonferroni correction = 0.05/5 outcomes = 0.01; CI is confidence interval

^a^ All continuous outcomes, exposures and controls were standardized and β was the standardized effect size

Use of strengths was prospectively inversely linked to the risk of depression diagnosis and positively with subsequent self-assessments of mental health. Increase in the use of strengths by one standard deviation was associated with a 38% reduction in odds of depression (OR = 0.619, 95% CI 0.481, 0.797) and an increase by 0.061 standard deviation in self-assessed mental health (β = 0.061, 95% CI 0.010, 0.113). The character strength of kindness was found to be positively associated with subsequent self-assessments of mental health (β = 0.059, 95% CI 0.013, 0.104) and with a 21% reduced odds of depression (OR = 0.793, 95% CI 0.633, 0.993). Delayed gratification was found to be prospectively inversely associated with the odds of diagnosed depression by 28% (OR = 0.721, 95% CI 0.573, 0.908) and of diagnosed anxiety by 20% (OR = 0.797, 95% CI 0.650, 0.976). Four of these associations did not pass the threshold of *p* < 0.05 after the correction for multiple testing.

Regarding the SMC-WBA scale, it was found to be positively associated with self-reported mental health (β = 0.118, 95% CI 0.048, 0.188) and with a 51% reduced odds of depression (OR = 0.487, 95% CI 0.350, 0.678).

### Strengths of moral character and physical health

There was a positive prospective association between the use of strengths indicator and subsequent self-assessments of physical health (β = 0.084, 95% CI 0.021, 0.136) (Table 3, right panel). The use of strengths was also found to be associated with reduced risk of subsequent cardiovascular disease by 61% with each standard deviation of the use of strengths measure (OR = 0.389, 95% CI 0.186, 0.811). However, the significance level for this association was below *p* < 0.05 after correction for multiple testing.

Orientation to promote good was positively associated with the subsequent self-assessed physical health. In particular, it was found to be associated with prior responses to the question ‘I always act to promote good in all circumstances, even in difficult and challenging situations’ (β = 0.076, 95% CI 0.028, 0.125). Positive prospective association was also found for the self-reports of physical health and delayed gratification (β = 0.048, 95% CI 0.000, 0.095); however, the significance level for the last association did not pass the threshold of *p* < 0.05 after the correction for multiple testing. Finally, the aggregate measure of strengths of character SMC-WBA was found to be prospectively positively associated with self-reports of physical health (β = 0.094, 95% CI 0.025, 0.163).

No significant effects for the associations between moral compass and kindness and subsequent self-reported physical health and diagnostic information on physical health outcomes were found.

### Robustness analysis

For the three regressions evaluating depression, anxiety, and a cardiovascular disease, the results obtained on a limited sample of respondents who did not have any history of disease related to the examined health outcome, were very similar to those obtained analyzing the entire sample (Table A2 in the Supplementary Information). When controlling for the alternate measure of 2018 overall well-being (Table A3 in the Supplementary Information) and simultaneously for five domain-specific scores in 2018 (Table A4 in the Supplementary Information), directionality of most associations was preserved but effects sizes were somewhat attenuated, and with wider confidence intervals. Nevertheless, one of the three indicators of orientation to promote good remained positively prospectively associated with lower risk of depression and the use of strengths item—with lower risk of cardiovascular disease. In addition, when comparing results from complete-case analyses to those from the core (multiply imputed) analyses—results were also very similar (Table A5 in the Supplementary Information). Next, the supplementary analyses with the use of limited set of controls (Table A6 in the Supplementary Information) showed that the pattern of significant associations remained the same with very comparable effect sizes. The only differences were noted for one temporal association with diagnosed anxiety (one estimate with the controls reflecting social determinants of health became significant) and another one with diagnosed cardiovascular disease (the only significant estimate became insignificant when a limited set of controls was used). Finally, in models with all indicators of SMC inserted concurrently, most associations were confirmed, even if slightly attenuated. Specifically, one indicator of orientation to promote good remained positively associated with subsequent self-reported physical health, the use of strengths item remained significantly associated with a lower risk of cardiovascular disease and higher self-reported physical health, and the indicator of delayed gratification was found to be associated with subsequent lower risk of anxiety (Table A7 in the Supplementary Information). This provided further evidence that the respective associations presented in the primary analyses were rather stable. In addition, the effect sizes in the supplementary analyses with the limited sets of controls were generally larger that these presented in the primary analyses, which implied that the findings are rather conservative and do not overestimate the prospective associations between strengths of character and diagnosed depression, as well as self-reports of mental and physical health.

The robustness of the results, assessed through the sensitivity measures E-values, provided additional evidence on at least modest robustness to unmeasured confounding of the examined associations (Table 4). Particularly, robust associations were those between using character strength and cardiovascular disease (*E* value = 4.58) and between strengths of character and depression (*E* value = 3.53).

**Table 4:** *E* values for effect measures and for CI limits for associations between strengths of moral character and subsequent health (survey data 2018–2019 merged with health insurance data 2017–2019, United States, *N* = 1209)

Strengths of moral character (0–10)	Mental health outcomes						Physical health outcomes			
	Self-reported mental health		Anxiety		Depression		Self-reported physical health		Cardiovascular disease	
	Effect estimate†	CI limit‡	Effect estimate†	CI limit‡	Effect estimate†	CI limit‡	Effect estimate†	CI limit‡	Effect estimate†	CI limit‡
Moral compass										
I always know the right thing to do	–	–	–	–	2.24	1.57	–	–	–	–
Orientation to promoting good										
I am willing to face difficulties in order to do what is right	1.31	1.13	–	–	2.20	1.68	–	–	–	–
I give up personal pleasures whenever it is possible to do some good instead	1.26	1.04	–	–	2.57	1.82	–	–	–	–
I always act to promote good in all circumstances, even in difficult and challenging situations	1.37	1.21	–	–	2.06	1.38	1.35	1.19	–	–
Use of strengths										
I get to use my strengths to help others (0–10)	1.30	1.10	–	–	2.61	1.82	1.37	1.20	4.58	1.77
Kindness										
I always treat everyone with kindness, fairness and respect	1.30	1.12	–	–	1.83	1.09	–	–	–	–
Delayed gratification										
I am always able to give up some happiness now for greater happiness later	–	–	1.82	1.18	2.12	1.44	1.26	1.02	–	–
Strengths of moral character scale (SMC-WBA)	1.47	1.26	–	–	3.53	2.31	1.40	1.18	–	–

See VanderWeele and Ding [58] for the formula for calculating E-values

^†^The *E* values for effect estimates are the minimum strength of association on the risk ratio scale that an unmeasured confounder would need to have with both the exposure and the outcome to fully explain away the observed association between the exposure and outcome, conditional on the measured covariates. For example, in the studied population an unmeasured confounder would need to be associated with both using one’s strengths to help others and cardiovascular disease by risk ratios of 4.58 each, above and beyond the measured covariates, to fully explain away the observed association between the two variables

^‡^The *E* values for the limit of the 95% confidence interval (CI) closest to the null denote the minimum strength of association on the risk ratio scale that an unmeasured confounder would need to have with both the exposure and the outcome to shift the confidence interval to include the null value, conditional on the measured covariates. For example, in the studied population, an unmeasured confounder would need to be associated with both using one’s strengths to help others and cardiovascular disease by 1.77-fold each, above and beyond the measured covariates, to shift the upper limit of the confidence interval to include the null

## Discussion

In the pursuit of identifying positive health stimuli, this study examined the links between SMC and health outcomes and identified at least five potential pathways for SMC’s contribution to health. First, the results suggest that persons who live their lives according to the moral compass have substantially lower odds of depression. This may be connected to the brain responses associated with the moral aspect of decision-making. Based on data from neuroimaging, during the decision-making process the most activated region of decision maker’s brain is the ventromedial prefrontal cortex [60]. The same region is involved in the activation and regulation of emotions in the situation of moral judgment [61]. Our study provided some indications that emotional processing of one’s own moral dilemma may contribute to mental health. Second, those who (1) act to promote and do good even at their own expense, while facing difficulties, as well as those who (2) perform acts of kindness, have higher subsequent self-reports of mental health and of physical health (the latter only for acts of kindness) as well as lower odds of depression. These paths corroborate evolutionary theories indicating that altruistic behaviors and the capacity for generosity contribute to enhanced social cooperation and strengthen adaptation to changing environment. Hence, they are believed to be conducive to the survival of humankind in the process of evolution. They are also believed to lead to positive, pleasurable feelings (i.e., happiness, optimism, self-confidence, feeling in control [62, 63] which have been shown, in turn, to be associated with better mental and physical health outcomes as well as longevity [64–66]. Third, use of SMC to help others in daily life was found to be prospectively associated with lower risk of depression and greater self-reported mental health and physical health, as well as lower risk of a cardiovascular disease. The reasons for this may be linked with the philosophical conviction, supported by some empirical evidence, that possession of positive character strengths contributes to well-being but only their habituation and exercising leads to genuine accomplishment and feeling of meaning in life, thus flourishing [1, 67, 68]. Finally, the results were also indicative of the protective role of delayed gratification (i.e., always being able to give up some happiness now for greater happiness later) against depression, possibly anxiety and higher self-reports of physical health. This path corresponds to prior research on predicting and understanding decisions people make when faced with immediate and delayed outcomes. They showed that present rewards are usually preferred over later gratifications [69]. However, preferences for delayed gratification can be relevant for generating positive health outcomes. In health-related choices involving delayed gratification very often the value of future incentives exceeds the value of immediate rewards [70]. For example, if one can refrain from immediate pleasurable activities (e.g., smoking a cigarette that gives some instant relief from a craving or helps alleviate stress), she can expect a greater future award, that is, a healthier outcome (e.g., lower risk of a lung cancer).

This study adds to the literature in the following ways. First, to the best of our knowledge, this is the first study that provides evidence for longitudinal associations between SMC and physical health outcomes. Specifically, contrary to prior cross-sectional evidence of no correlation between application of character strengths and self-reports of physical health [7], this work provides at least modest empirical evidence that possessing and using SMC may be beneficial for one’s physical health (for both self-reported assessment of one’s physical health as well as cardiovascular disease prevention). In this vein, our evidence is also in line with the recent findings on the usefulness of strength-based interventions in pain self-efficacy and the capacity to function despite pain [16] and in older age [15]. Regarding the mental health outcomes, our results corroborate the earlier evidence from experimental studies [14, 25, 71], meta-analyses [9, 17, 72–74] and longitudinal observational studies [15] that SMC and their use may provide benefits especially for emotional well-being. Conversely, our results challenge the experimental findings of Khanna and Singh [24] who reported no contribution of some strengths of character to decreased depressive symptoms. In addition, our results regarding the association between the delayed gratification and health outcomes are in line with prior research on the usefulness of delayed gratification for predicting health behaviors [70]. Second, using multiple health outcomes, both self-reported and derived from the health insurance administrative data, this study exposes some patterns of associations between SMC and health that might not have been discernible if single outcomes were examined in separate studies. Specifically, it shows that the temporal associations are more pronounced for mental health than for physical health. Third, the longitudinal design and the adjustment for an extensive range of covariates, prior values of the exposure (in the case of medical claims outcomes) and of the baseline outcomes, helped to establish a temporal association and to strengthen evidence against reverse causation and unmeasured confounding. Finally, sensitivity analyses for unmeasured confounding and a series of secondary analyses provided supporting evidence in favor of robustness of our results. Regarding the re-estimation of the primary regression models with two additional sets of controls we note, however, that using overall well-being at baseline we might over-control, especially if the aspects of moral character are relatively stable (although in our sample we observed significant increases in each indicator of strengths of moral character between wave 1 and wave 2, Table 2) and either have already exerted some of their effects on health or still require more time to affect health. If this is the case, our control for baseline well-being, next to other controls and health outcomes at baseline, was essentially blocking some of these associations. Consequently, existing associations might not have been detected. This might be the case in our supplementary analysis, as previous analysis on the three-wave dataset provided evidence of the predictive character of an orientation to promote good for the well-being outcomes [6].

Despite its strengths, this study is also subject to certain limitations. First, this study did not use an experimental design that is a gold standard in establishing causal relationships. Second, since our study used self-reports of SMC and of two health outcomes, it may be subject to social desirability bias [75] and consequently report results of limited accuracy and reliability. However, the longitudinal design and controlling for baseline outcomes limits this bias. Third, although the study was designed to rely on random sampling for the selection of survey participants, eventually only self-selected working adults, mostly white-collar workers, provided data for the analyses. Although our sample was not substantially different from the targeted population in terms of major demographic variables, further analyses should be performed to replicate the results in different populations. Likewise, attrition between waves 1 and 2 may constitute another concern. Fourth, the follow-up period in this study was relatively short (1 year), which might be insufficient to observe changes in the level of character strengths as well as the effects of accumulation of character strengths, especially on physical health. However, there are some recent evidence that strengths such as humor, spirituality and prudence might be malleable even in a relatively short period of time [26, 76]. Finally, there could be some criticism that one of the more recognized VIA character strength instruments by Seligman and Peterson [36] and VIA Institute on Character (https://www.viacharacter.org) was not used. While we recognize that these instruments are available and have been frequently applied, it was our intention to develop and use a short measure that drew upon long-standing traditions in the humanities, focuses on SMC, is comprehensive and reliable to measure well-being and that would be well suited for inclusion in workplace surveys that address complete well-being [4, 29, 33].

## Supplementary Information

Below is the link to the electronic supplementary material.

Supplementary file1 (DOCX 66 kb)

## Data Availability

The dataset generated during and/or analyzed during the current study is available from the corresponding author on reasonable request.

## Footnotes

1 Well-Being Assessment (WBA) ﻿is a freely available instrument to measure complete well-being in six domains: emotional health, physical health, meaning and purpose, character strengths, social connectedness, and financial security. Either the overall WBA score or a single well-being domain score (corresponding to a subset of indicators of the domain of interest) can be used. The WBA instrument as well as of each of its domains demonstrate satisfactory psychometric properties.
