# Supplementary material for: Prospective associations between strengths of moral character and health: longitudinal evidence from survey and insurance claims data
Source: Soc Psychiatry Psychiatr Epidemiol. 2022 Aug 2;58(1):163–76. doi: 10.1007/s00127-022-02344-5 (PMC9344441; doi:10.1007/s00127-022-02344-5)
Supplement: Supplementary file 1 — Supplementary file1 (DOCX 66 kb) [file 127_2022_2344_MOESM1_ESM.docx]

**Prospective associations between strengths of moral character and health. Longitudinal evidence from survey and insurance claims data**

Supplementary Information

**Table A1**

*Zero-order correlations between study variables at baseline (Well-Being Survey and health insurance administrative data); United States, 2017-2019).*

| Variable | (1) | (2) | (3) | (4) | (5) | (6) | (7) | (8) | (9) | (10) | (11) | (12) |
| --- | --- | --- | --- | --- | --- | --- | --- | --- | --- | --- | --- | --- |
| I always know the right thing to do (1) |  |  |  |  |  |  |  |  |  |  |  |  |
| I am willing to face difficulties in order to do what is right (2) | 0.43*** |  |  |  |  |  |  |  |  |  |  |  |
| I give up personal pleasures whenever it is possible to do some good instead (3) | 0.48*** | 0.55*** |  |  |  |  |  |  |  |  |  |  |
| I always act to promote good in all circumstances, even in difficult and challenging situations (4) | 0.36*** | 0.57*** | 0.48*** |  |  |  |  |  |  |  |  |  |
| I get to use my strengths to help others (5) | 0.37*** | 0.49*** | 0.47*** | 0.50*** |  |  |  |  |  |  |  |  |
| I always treat everyone with kindness, fairness and respect (6) | 0.39*** | 0.49*** | 0.61*** | 0.43*** | 0.41*** |  |  |  |  |  |  |  |
| I am always able to give up some happiness now for greater happiness later (7) | 0.36*** | 0.59*** | 0.48*** | 0.57*** | 0.46*** | 0.47*** |  |  |  |  |  |  |
| Strengths of moral character scale (SMC-WBA) (8) | 0.66*** | 0.78*** | 0.78*** | 0.77*** | 0.72*** | 0.71*** | 0.76*** |  |  |  |  |  |
| Self-reported mental health (9) | 0.39*** | 0.39*** | 0.46*** | 0.28*** | 0.39*** | 0.29*** | 0.36*** | 0.49*** |  |  |  |  |
| Self-reported physical health (10) | 0.24*** | 0.22*** | 0.29*** | 0.20*** | 0.25*** | 0.16*** | 0.26*** | 0.32*** | 0.49*** |  |  |  |
| Diagnosis of anxiety (11) | -0.04 | -0.07 | -0.04 | -0.04 | -0.01 | -0.01 | -0.08** | -0.05 | -0.25*** | -0.13*** |  |  |
| Diagnosis of depression (12) | -0.12*** | -0.11*** | -0.14*** | -0.08** | -0.08** | -0.06 | -0.15*** | -0.14*** | -0.28*** | -0.16*** | 0.34***^a^ |  |
| Diagnosis of cardiovascular disease (13) | 0.02 | 0.03 | 0.02 | 0.03 | 0.03 | 0.01 | 0.03 | 0.03 | 0.02 | -0.01 | 0.05 | 0.06*^a^ |

**** p<0.001, ** p<0.01, * p<0.05;* *Pearson’s correlation coefficient is used as a measure of association between variables (1) – (10); Point biserial correlation coefficient is reported as a measure of association between dichotomous variables (11)-(13) and continuous variables (1) – (10); Pearson’s phi coefficient was reported as a measure of correlation between dichotomous variables (11)-(13);^a^ significance level reported based on the chi-square test;*

**Table A2**

*Associations between strengths of moral character and subsequent health (Well-Being Survey and health insurance administrative data); United States, 2017-2019, limited sample)^a^. For depression, anxiety and cardiovascular disease outcomes, sample was limited to those who did not experience the health outcome under scrutiny prior to the exposure.*

| **Strengths of moral character (0-10)** | Mental health outcome | | | Physical health outcome | |
| --- | --- | --- | --- | --- | --- |
|  | Self-reported mental health (N=1,209, the same as in Table 3) | Anxiety^c^  (N=1,053) | Depression^c^  (N=1,093) | Self-reported physical health  (N=1,209, the same as in Table 3) | Cardiovascular disease^c^  (N=701) |
|  | β ^b^  95% CI | OR  95% CI | OR  95% CI | β ^b^  95% CI | OR  95% CI |
| ***Moral compass*** |  |  |  |  |  |
| I always know the right thing to do | 0.019  (-0.028, 0.067) | 0.792  (0.621, 1.012) | 0.644***  (0.492, 0.842) | 0.019  (-0.028, 0.067) | 1.375  (0.588, 3.212) |
| ***Orientation to promote good*** |  |  |  |  |  |
| I am willing to face difficulties in order to do what is right | 0.062*  (0.014, 0.109) | 0.899  (0.691, 1.169) | 0.684**  (0.523, 0.895) | 0.039  (-0.009, 0.087) | 0.949  (0.413, 2.178) |
| I give up personal pleasures whenever it is possible to do some good instead | 0.048**  (0.002, 0.095) | 0.889  (0.682, 1.158) | 0.596***  (0.450, 0.791) | 0.027  (-0.021, 0.075) | 1.131  (0.497, 2.571) |
| I always act to promote good in all circumstances, even in difficult and challenging situations | 0.083***  (0.034, 0.132) | 0.941  (0.724, 1.222) | 0.696**  (0.529, 0.916) | 0.076**  (0.028, 0.125) | 1.541  (0.589, 4.003) |
| ***Use of strengths*** |  |  |  |  |  |
| I get to use my strengths to help others | 0.061**  (0.010, 0.113) | 0.977  (0.736, 1.298) | 0.584***  (0.435, 0.784) | 0.084**  (0.021, 0.136) | 0.369**  (0.155, 0.877) |
| ***Kindness*** |  |  |  |  |  |
| I always treat everyone with kindness, fairness and respect | 0.059*  (0.013, 0.104) | 1.046  (0.809, 1.353) | 0.777*  (0.604, 0.999) | 0.034  (-0.012, 0.080) | 1.300  (0.537, 3.151) |
| ***Delayed gratification*** |  |  |  |  |  |
| I am always able to give up some happiness now for greater happiness later | 0.046  (-0.001, 0.094) | 0.930  (0.720, 1.202) | 0.726*  (0.557,0.946) | 0.048*  (0.000, 0.095) | 1.780  (0.725, 4.469) |
| ***Strengths of moral character scale (SMC-WBA)*** | 0.117***  (0.047, 0.187) | 0.844  (0.585, 1.217) | 0.452***  (0.308, 0.663) | 0.094**  (0.025, 0.163) | 1.146  (0.365, 3.609) |

*** p<0.001, ** p<0.01, * p<0.05; the p value cut-off for Bonferroni correction = 0.05/5 outcomes = 0.01; CI is confidence interval.

^a^ A set of regression models was used to regress each outcome on each character strength exposure separately, to estimate odds ratio (OR) for binary outcomes or β (where the outcome is continuous). Each analysis was controlled for demographics (gender, age, race, education, marital status, having children at home, taking care of an elderly), wealth and income (home ownership and salary), lifestyle (voting in the last elections, religious service attendance, spiritual practices, volunteering, community work) and work characteristics (number of work hours, supervisor support, job control, job demand and job meaning). These variables were controlled for in the first wave (in the same wave as the exposure), since only two waves of survey data were available. Additionally, in each regression the number of diagnosed health conditions (ranging from 0 to 37 possible diagnosed health conditions) prior to exposure was used as a control.

^b^ All continuous outcomes, exposures and controls were standardized and β was the standardized effect size.

^c^ Sample limited to those who did not experience the health outcome under scrutiny prior to exposure.

**Table A3**

*Associations between strengths of moral character and subsequent health (Well-Being Survey, health insurance administrative data and an additional control for the alternate overall 2018 well-being index; without the strengths of character domain^a^; United States, 2017-2019, N=1,209).*

| **Strengths of moral character (0-10)** | Mental health outcome | | | Physical health outcome | |
| --- | --- | --- | --- | --- | --- |
|  | Self-reported mental health | Anxiety | Depression | Self-reported physical health | Cardiovascular disease |
|  | β ^b^  95% CI | OR  95% CI | OR  95% CI | β ^b^  95% CI | OR  95% CI |
| ***Moral compass*** |  |  |  |  |  |
| I always know the right thing to do | -0.025  (-0.075, 0.024) | 0.999  (0.802, 1.245) | 0.862  (0.677, 1.099) | -0.053  (-0.107, 0.001) | 1.026  (0.504, 2.083) |
| ***Orientation to promote good*** |  |  |  |  |  |
| I am willing to face difficulties in order to do what is right | 0.025  (-0.025, 0.075) | 0.981  (0.784, 1.227) | 0.906  (0.706, 1.162) | -0.036  (-0.090, 0.018) | 0.840  (0.403, 1.751) |
| I give up personal pleasures whenever it is possible to do some good instead | -0.010  (-0.059, 0.039) | 1.039  (0.825, 1.309) | 0.752*  (0.581, 0.973) | -0.002  (-0.055, 0.050) | 1.011  (0.504, 2.027) |
| I always act to promote good in all circumstances, even in difficult and challenging situations | 0.048  (-0.002, 0.100) | 1.112  (0.877, 1.410) | 1.009  (0.781, 1.303) | 0.010  (-0.046, 0.066) | 1.456  (0.627, 3.384) |
| ***Use of strengths*** |  |  |  |  |  |
| I get to use my strengths to help others | -0.031  (-0.086, 0.025) | 1.168  (0.911, 1.497) | 0.849  (0.640, 1.126) | 0.006  (-0.054, 0.066) | 0.324 *  (0.132, 0.789) |
| ***Kindness*** |  |  |  |  |  |
| I always treat everyone with kindness, fairness and respect | 0.020  (-0.027, 0.067) | 1.113  (0.891, 1.391) | 0.992  (0.780, 1.261) | -0.013  (-0.064, 0.038) | 0.971  (0.457, 2.063) |
| ***Delayed gratification*** |  |  |  |  |  |
| I am always able to give up some happiness now for greater happiness later | -0.005  (-0.050, 0.049) | 0.880  (0.707, 1.096) | 0.935  (0.726,1.205) | 0.006  (-0.047, 0.059) | 1.308  (0.584, 2.931) |
| ***Strengths of moral character scale (SMC-WBA)*** | 0.010  (-0.066, 0.087) | 1.081  (0.760, 1.538) | 0.783  (0.533, 1.149) | -0.031  (-0.114, 0.053) | 0.825  (0.267, 2.549) |

*** p<0.001, ** p<0.01, * p<0.05; the p value cut-off for Bonferroni correction = 0.05/5 outcomes = 0.01; CI is confidence interval.

^a^ A set of regression models was used to regress each outcome on each character strength exposure separately, to estimate odds ratio (OR) for binary outcomes or β (for continuous outcomes). Each analysis was controlled for demographics (gender, age, race, education, marital status, having children at home, taking care of an elderly), wealth and income (home ownership and salary), lifestyle (voting in the last elections, religious service attendance, spiritual practices, volunteering, community work) and work characteristics (number of work hours, supervisor support, job control, job demand and job meaning). These variables were controlled for in the first wave (in the same wave as the exposure), since only two waves of survey data were available. Additionally, in each regression (with an exception of self-reported physical health and mental health outcomes) an outcome prior to exposure as well as the number of diagnosed health conditions (ranging from 0 to 37 possible diagnosed health conditions) prior to exposure were applied as controls. Finally, controlling for all of the well-being index (net strengths of character domain) scores in 2018 was applied

^b^ All continuous outcomes, exposures and controls were standardized and β was the standardized effect size.

**Table A4**

*Associations between strengths of moral character and subsequent health (Well-Being Survey, health insurance administrative data and additionally, controlling for all of the five separate well-being domain-specific scores; without character strength domain, United States, 2017-2019, N=1,209)^a^.*

| **Strengths of moral character (0-10)** | Mental health outcome | | | Physical health outcome | |
| --- | --- | --- | --- | --- | --- |
|  | Self-reported mental health | Anxiety | Depression | Self-reported physical health | Cardiovascular disease |
|  | β ^b^  95% CI | OR  95% CI | OR  95% CI | β ^b^  95% CI | OR  95% CI |
| ***Moral compass*** |  |  |  |  |  |
| I always know the right thing to do | -0.031  (-0.080, 0.018) | 1.013  (0.808, 1.267) | 0.776  (0.586, 1.027) | -0.034  (-0.089, 0.020) | 0.978  (0.443, 2.157) |
| ***Orientation to promote good*** |  |  |  |  |  |
| I am willing to face difficulties in order to do what is right | 0.009  (-0.040, 0.059) | 1.018  (0.810, 1.278) | 0.907  (0.704, 1.169) | -0.026  (-0.079, 0.027) | 0.739  (0.321, 1.700)) |
| I give up personal pleasures whenever it is possible to do some good instead | -0.006  (-0.054, 0.042) | 1.040  (0.822, 1.315) | 0.734*  (0.564, 0.955) | 0.004  (-0.048, 0.055) | 1.072  (0.510, 2.257) |
| I always act to promote good in all circumstances, even in difficult and challenging situations | 0.024  (-0.028, 0.076) | 1.168  (0.913, 1.496) | 1.014  (0.778, 1.322) | 0.036  (-0.020, 0.092) | 1.182  (0.437, 3.200) |
| ***Use of strengths*** |  |  |  |  |  |
| I get to use my strengths to help others | -0.017  (0.073, 0.038) | 1.176  (0.909, 1.522) | 0.812  (0.606, 1.087) | 0.031  (-0.029, 0.092) | 0.263*  (0.088, 0.794) |
| ***Kindness*** |  |  |  |  |  |
| I always treat everyone with kindness, fairness and respect | 0.016  (-0.030, 0.062) | 1.138  (0.907, 1.428) | 0.979  (0.770, 1.246) | -0.008  (-0.057, 0.042) | 0.948  (0.400, 2.251) |
| ***Delayed gratification*** |  |  |  |  |  |
| I am always able to give up some happiness now for greater happiness later | 0.000  (-0.048, 0.048) | 0.886  (0.709, 1.107) | 0.925  (0.714, 1.199) | 0.013  (-0.039, 0.065) | 1.168  (0.470, 2.907) |
| ***Strengths of moral character scale (SMC-WBA)*** | 0.001  (-0.077, 0.079) | 1.133  (0.785, 1.634) | 0.751  (0.502, 1.124) | 0.003  (-0.081, 0.087) | 0.696  (0.128, 2.659) |

*** p<0.001, ** p<0.01, * p<0.05; the p value cut-off for Bonferroni correction = 0.05/5 outcomes = 0.01; CI is confidence interval.

^a^ A set of regression models was used to regress each outcome on each character strength exposure separately, to estimate odds ratio (OR) for binary outcomes or β (for continuous outcomes). Each analysis was controlled for demographics (gender, age, race, education, marital status, having children at home, taking care of an elderly), wealth and income (home ownership and salary), lifestyle (voting in the last elections, religious service attendance, spiritual practices, volunteering, community work) and work characteristics (number of work hours, supervisor support, job control, job demand and job meaning). These variables were controlled for in the first wave (in the same wave as the exposure), since only two waves of survey data were available. Additionally, in each regression (with an exception of self-reported physical health and mental health outcomes) an outcome prior to exposure as well as the number of diagnosed health conditions (ranging from 0 to 37 possible diagnosed health conditions) prior to exposure were applied as controls. Finally, controlling for all of the 5 separate well-being domain-specific scores (excluding strengths of character domain) in 2018 was applied.

^b^ All continuous outcomes, exposures and controls were standardized and β was the standardized effect size.

**Table A5**

*Associations between strengths of moral character and subsequent health (Well-Being Survey and health insurance administrative data; United States, 2017-2019, full case scenario)^a^.*

| **Strengths of moral character (0-10)** | Mental health outcome | | | Physical health outcome | |
| --- | --- | --- | --- | --- | --- |
|  | Self-reported mental health (N=1,116) | Anxiety  (N=1,119) | Depression  (N=1,119) | Self-reported physical health  (N=1,105) | Cardiovascular disease  (N=1,027) |
|  | β ^b^  95% CI | OR  95% CI | OR  95% CI | β ^b^  95% CI | OR  95% CI |
| ***Moral compass*** |  |  |  |  |  |
| I always know the right thing to do | 0.017  (-0.033, 0.067) | 0.854  (0.690, 1.057) | 0.661**  (0.519, 0.841) | 0.012  (-0.036, 0.061) | 0.935  (0.457, 1.915) |
| ***Orientation to promote good*** |  |  |  |  |  |
| I am willing to face difficulties in order to do what is right | 0.068**  (0.018, 0.119) | 0.852  (0.688, 1.056) | 0.655**  (0.512, 0.837) | 0.043  (-0.005, 0.019) | 0.793  (0.399, 1.576) |
| I give up personal pleasures whenever it is possible to do some good instead | 0.055**  (0.006, 0.103) | 0.898  (0.719, 1.122) | 0.639**  (0.496, 0.822) | 0.024  (-0.025, 0.072) | 1.024  (0.512, 2.051) |
| I always act to promote good in all circumstances, even in difficult and challenging situations | 0.083**  (0.032, 0.134) | 0.966  (0.776, 1.203) | 0.748*  (0.588, 0.952) | 0.074**  (0.025, 0.124) | 1.319  (0.583, 2.984) |
| ***Use of strengths*** |  |  |  |  |  |
| I get to use my strengths to help others | 0.055**  (0.000, 0.107) | 1.004  (0.793, 1.270) | 0.637**  (0.488, 0.831) | 0.075**  (0.021, 0.128) | 0.396*  (0.188, 0.853) |
| ***Kindness*** |  |  |  |  |  |
| I always treat everyone with kindness, fairness and respect | 0.053*  (0.005, 0.100) | 0.988  (0.794, 1.232) | 0.803  (0.632, 1.019) | 0.028  (-0.019, 0.076) | 0.941  (0.444, 1.993) |
| ***Delayed gratification*** |  |  |  |  |  |
| I am always able to give up some happiness now for greater happiness later | 0.046  (-0.003, 0.095) | 0.786*  (0.638, 0.970) | 0.700**  (0.549,0.892) | 0.043  (-0.005, 0.092) | 2.057  (0.776, 5.448) |
| ***Strengths of moral character scale (SMC-WBA)*** | 0.087**  (0.032; 0.142) | 0.853  (0.678; 1.073) | 0.585***  (0.450; 0.760) | 0.066*  (0.012; 0.119) | 0.830  (0.394, 1.751) |

*** p<0.001, ** p<0.01, * p<0.05; the p value cut-off for Bonferroni correction = 0.05/5 outcomes = 0.01; CI is confidence interval.

^a^ A set of regression models was used to regress each outcome on each character strength exposure separately, to estimate odds ratio (OR) for binary outcomes or β (for continuous outcomes). Each analysis was controlled for demographics (gender, age, race, education, marital status, having children at home, taking care of an elderly), wealth and income (home ownership and salary), lifestyle (voting in the last elections, religious service attendance, spiritual practices, volunteering, community work) and work characteristics (number of work hours, supervisor support, job control, job demand and job meaning). These variables were controlled for in the first wave (in the same wave as the exposure), since only two waves of survey data were available. Additionally, in each regression an outcome prior to exposure as well as the number of diagnosed health conditions (ranging from 0 to 37 possible diagnosed health conditions) prior to exposure were applied as controls.

^b^ All continuous outcomes, exposures and controls were standardized and β was the standardized effect size.

**Table A6**

*Associations between strengths of moral character and subsequent health – different sets of controls (Well-Being Survey and health insurance administrative data; United States, 2017-2019, N=1,209)^a^*

| **Strengths of moral character (0-10)** | Model^c^ | Mental health outcome | | | Physical health outcome | |
| --- | --- | --- | --- | --- | --- | --- |
|  |  | Self-reported mental health | Anxiety | Depression | Self-reported physical health | Cardiovascular disease |
|  |  | β ^b^  95% CI | OR  95% CI | OR  95% CI | β ^b^  95% CI | OR  95% CI |
| ***Moral compass*** |  |  |  |  |  |  |
| I always know the right thing to do | Model 1 | 0.034  (-0.012; 0.081) | 0.820*  (0.678; 0.992) | 0.703**  (0.568; 0.870) | 0.033  (-0.014; 0.079) | 1.092  (0.628; 1.900) |
|  | Model 2 | 0.033  (-0.014; 0.080) | 0.862  (0.709; 1.048) | 0.709**  (0.571; 0.881) | 0.033  (-0.014; 0.080) | 1.229  (0.663; 2.277) |
|  | Model 3 | 0.019  (-0.028, 0.067) | 0.881  (0.719, 1.077) | 0.694***  (0.554, 0.869) | 0.019  (-0.028, 0.067) | 0.976  (0.484, 1.967) |
| ***Orientation to promote good*** |  |  |  |  |  |  |
| I am willing to face difficulties in order to do what is right | Model 1 | 0.082**  (0.035; 0.128) | 0.879  (0.729; 1.061) | 0.732**  (0.591; 0.906) | 0.061**  (0.015; 0.107) | 1.165  (0.688; 1.974) |
|  | Model 2 | 0.077**  (0.030; 0.124) | 0.886  (0.730; 1.074) | 0.725**  (0.582; 0.903) | 0.057*  (0.011; 0.104) | 1.104  (0.638; 1.910) |
|  | Model 3 | 0.062*  (0.014, 0.109) | 0.866  (0.706, 1.063) | 0.703**  (0.512, 0.837) | 0.039  (-0.009, 0.087) | 0.795  (0.399, 1.582) |
| I give up personal pleasures whenever it is possible to do some good instead | Model 1 | 0.063**  (0.018; 0.109) | 0.905  (0.742; 1.104) | 0.653***  (0.521; 0.817) | 0.039  (-0.007; 0.085) | 1.209  (0.670; 1.182) |
|  | Model 2 | 0.060*  (0.013; 0.106) | 0.932  (0.757; 1.147) | 0.639***  (0.505; 0.808) | 0.039  (-0.008; 0.086) | 1.157  (0.636; 2.106) |
|  | Model 3 | 0.048**  (0.002, 0.095) | 0.930  (0.749, 1.155) | 0.626***  (0.492, 0.798) | 0.027  (-0.021, 0.075) | 0.957  (0.482, 1.900 |
| I always act to promote good in all circumstances, even in difficult and challenging situations | Model 1 | 0.106***  (0.059; 0.154) | 0.900  (0.745; 1.085) | 0.769*  (0.634; 0.946) | 0.094***  (0.048; 0.140) | 1.578  (0.852; 2.924) |
|  | Model 2 | 0.102***  (0.055; 0.150) | 0.915  (0.753; 1.111) | 0.754*  (0.608; 0.935) | 0.092***  (0.045; 0.138) | 1.783  (0.914; 3.481) |
|  | Model 3 | 0.083***  (0.034, 0.132) | 0.927  (0.752, 1.142) | 0.735**  (0.584, 0.925) | 0.076**  (0.028, 0.125) | 1.266  (0.562, 2.849) |
| ***Use of strengths*** |  |  |  |  |  |  |
| I get to use my strengths to help others | Model 1 | 0.094***  (0.047; 0.141) | 0.951  (0.786; 1.150) | 0.680***  (0.551; 0.839) | 0.100***  (0.054; 0.146) | 0.985  (0.601; 1.616) |
|  | Model 2 | 0.085***  (0.037; 0.133) | 1.010  (0.828; 1.233) | 0.692***  (0.556; 0.861) | 0.097***  (0.050; 0.145) | 0.778  (0.468; 1.293) |
|  | Model 3 | 0.061**  (0.010, 0.113) | 0.986  (0.786, 1.238) | 0.619***  (0.481, 0.797) | 0.084**  (0.021, 0.136) | 0.389*  (0.186, 0.811) |
| ***Kindness*** |  |  |  |  |  |  |
| I always treat everyone with kindness, fairness and respect | Model 1 | 0.075**  (0.031; 0.120) | 0.966  (0.791; 1.179) | 0.820  (0.662; 1.014) | 0.049*  (0.004; 0.094) | 1.172  (0.662; 2.074) |
|  | Model 2 | 0.076*  (0.031; 0.120) | 0.972  (0.794; 1.189) | 0.795*  (0.640; 0.989) | 0.048*  (0.003; 0.093) | 1.351  (0.711; 2.568) |
|  | Model 3 | 0.059*  (0.013, 0.104) | 1.002  (0.809, 1.240) | 0.793*  (0.633, 0.993) | 0.034  (-0.012, 0.080) | 0.940  (0.444, 1.991) |
| ***Delayed gratification*** |  |  |  |  |  |  |
| I am always able to give up some happiness now for greater happiness later | Model 1 | 0.069**  (0.023; 0.115) | 0.800*  (0.663; 0.964) | 0.756**  (0.611; 0.936) | 0.068**  (0.022; 0.114) | 1.574  (0.872; 2.838) |
|  | Model 2 | 0.067**  (0.021; 0.114) | 0.799*  (0.661; 0.966) | 0.747**  (0.601; 0.927) | 0.066**  (0.019; 0.112) | 1.691  (0.891; 3.208) |
|  | Model 3 | 0.046  (-0.001, 0.094) | 0.797*  (0.650, 0.976) | 0.721**  (0.573, 0.908) | 0.048*  (0.000, 0.095) | 1.157  (0.545; 2.456) |
| ***Strengths of moral character scale  (SMC-WBA)*** | Model 1 | 0.156***  (0.090; 0.223) | 0.797  (0.615; 1.032) | 0.553***  (0.414; 0.740) | 0.123***  (0.059; 0.186) | 1.472  (0.674; 3.216) |
|  | Model 2 | 0.150***  (0.083; 0.218) | 0.830  (0.635; 1.085) | 0.544***  (0.403; 0.733) | 0.120***  (0.051; 0.185) | 1.477  (0.641; 3.404) |
|  | Model 3 | 0.118***  (0.048, 0.188) | 0.820  (0.609, 1.104) | 0.487***  (0.350, 0.678) | 0.096**  (0.027, 0.165) | 0.737  (0.270, 2.010) |

***p<0.001, ** p<0.01, * p<0.05; the p value cut-off for Bonferroni correction = 0.05/5 outcomes = 0.01; CI is confidence interval.

^a^ A set of regression models was used to regress each outcome on each character strength exposure separately, to estimate odds ratio (OR) for binary outcomes or β (for continuous outcomes). Each analysis was controlled for demographics (gender, age, race, education, marital status, having children at home, taking care of an elderly), wealth and income (home ownership and salary), lifestyle (voting in the last elections, religious service attendance, spiritual practices, volunteering, community work) and work characteristics (number of work hours, supervisor support, job control, job demand and job meaning). These variables were controlled for in the first wave (in the same wave as the exposure), since only two waves of survey data were available. Additionally, in each regression an outcome prior to exposure as well as the number of diagnosed health conditions (ranging from 0 to 37 possible diagnosed health conditions) prior to exposure were applied as controls.

^b^ All continuous outcomes, exposures and controls were standardized and β was the standardized effect size.

^c^ Model 1 – controlling for only social determinant of health (gender, age, race, educational attainment, marital status, having children at home, taking care of an elderly, wealth and income; Model 2 – controlling for social determinant of health + social participation and civic engagement (volunteering, community work, voting in the last elections, religious service attendance and spiritual practices); Model 3 – full set of controls (i.e., social determinant of health + social participation and civic engagement + work characteristics) as in the primary analyses presented here for the comparison purposes. All models also controlled for the history of disease to limit the risk of reverse causation.

**Table A7**

*Associations between strengths of moral character (individual items entered simultaneously) and subsequent health (Well-Being Survey and health insurance administrative data; United States, 2017-2019, N=1,209)^a^.*

| **Strengths of moral character (0-10)** | Mental health outcome | | | Physical health outcome | |
| --- | --- | --- | --- | --- | --- |
|  | Self-reported mental health | Anxiety | Depression | Self-reported physical health | Cardiovascular disease |
|  | β ^b^  95% CI | OR  95% CI | OR  95% CI | β ^b^  95% CI | OR  95% CI |
| ***Moral compass*** |  |  |  |  |  |
| I always know the right thing to do | -0.022  (-0.074, 0.030) | 0.901  (0.712, 1.140) | 0.801  (0.621, 1.035) | -0.020  (-0.075, 0.034) | 0.953  (0.405, 2.245) |
| ***Orientation to promote good*** |  |  |  |  |  |
| I am willing to face difficulties in order to do what is right | 0.026  (-0.035, 0.087) | 0.934  (0.710, 1.229) | 0.957  (0.699, 1.310) | -0.004  (-0.068, 0.059) | 0.385  (0.096, 1.543) |
| I give up personal pleasures whenever it is possible to do some good instead | 0.002  (-0.058, 0.061) | 1.075  (0.811, 1.426) | 0.752  (0.548, 1.031) | -0.028  (-0.089, 0.033) | 1.733  (0.553, 5.434) |
| I always act to promote good in all circumstances, even in difficult and challenging situations | 0.060  (-0.002, 0.123) | 0.973  (0.736, 1.287) | 0.995  (0.725, 1.365) | 0.075*  (0.011, 0.139) | 1.949  (0.645, 5.887) |
| ***Use of strengths*** |  |  |  |  |  |
| I get to use my strengths to help others | 0.031  (-0.027, 0.090) | 1.100  (0.840, 1.440) | 0.763  (0.566, 1.027) | 0.074*  (0.013, 0.134) | 0.256**  (0.105, 0.628) |
| ***Kindness*** |  |  |  |  |  |
| I always treat everyone with kindness, fairness and respect | 0.016  (-0.041, 0.072) | 1.164  (0.889, 1.526) | 1.057  (0.786, 1.422) | -0.018  (-0.077, 0.040) | 0.845  (0.301, 2.374) |
| ***Delayed gratification*** |  |  |  |  |  |
| I am always able to give up some happiness now for greater happiness later | 0.002  (-0.057, 0.061) | 0.759*  (0.581, 0.992) | 0.983  (0.724, 1.334) | 0.025  (-0.036, 0.086) | 2.579  (0.651, 10.212) |

*** p<0.001, ** p<0.01, * p<0.05; the p value cut-off for Bonferroni correction = 0.05/5 outcomes = 0.01; CI is confidence interval.

^a^ A set of regression models was used to regress each outcome on all character strength exposures simultaneously, to estimate odds ratio (OR) for binary outcomes or β (for continuous outcomes). Each analysis was controlled for demographics (gender, age, race, education, marital status, having children at home, taking care of an elderly), wealth and income (home ownership and salary), lifestyle (voting in the last elections, religious service attendance, spiritual practices, volunteering, community work) and work characteristics (number of work hours, supervisor support, job control, job demand and job meaning). These variables were controlled for in the first wave (in the same wave as the exposure), since only two waves of survey data were available. Additionally, in each regression an outcome prior to exposure as well as the number of diagnosed health conditions (ranging from 0 to 37 possible diagnosed health conditions) prior to exposure were applied as controls. Variance inflation factor for each item of strengths of moral character was below 2.2, indicating the multicollinearity was not an issue.

^b^ All continuous outcomes, exposures and controls were standardized and β was the standardized effect size.

**Note on similarities and dissimilarities between the strengths of moral character (SMC-WBA)** **and the VIA Survey of Character Strengths (Peterson and Seligman, 2004)**

The SMC-WBA is related to the measure of character strength developed by Peterson and Seligman, the VIA Survey of Character Strengths, that has been the focus of much empirical research (https://www.viacharacter.org; Peterson and Seligman, 2004). First, the MSC-WBA item *‘I always treat everyone with kindness, fairness and respect’* mentions two of the 24 VIA strengths by name *(‘kindness’* and *‘fairness’*), while *‘respect’* is mentioned in an item in the *‘citizenship’* VIA strength. Second, our item on delayed gratification (i.e., *‘I am always able to give up some happiness now for greater happiness later’*) assesses the *‘self-regulation’* VIA strength, and perhaps other strengths as well (a sample item from the VIA: *‘I never want things that are bad for me in the long run, even if they make me feel good in the short run’* shows this similarity). Third, our moral compass and orientation to promote good items relate to the VIA strength of ‘prudence’ and perhaps others. Fourth*,* our item*‘I get to use my strengths to help others’* refers to strengths in general (which could include both moral and intellectual strengths) as they are used in a moral manner to help others. The SMC-WBA differs from the VIA character strengths measure in terms of length (the SMC-WBA contains far fewer items, which is desirable when respondent time is limited), the number of strengths included (the SMC-WBA is focused on morality, whereas the VIA incorporates non-moral strengths), and includes a general item about the prosocial use of character strengths. In the workplace setting, in which our study was conducted, the VIA was too long given the focus of the survey on a variety of domains of well-being and we were especially interested in the effects of high standards of moral behavior [which might actually be a strength in workplaces and other settings as suggested by other scholars (Bagozzi et al., 2013; Comer and Vega, 2011; Sekerka and Bagozzi, 2007)], rather than a variety of character strengths.

**References**

Bagozzi, R.P., Sekerka, L.E., Hill, V., Sguera, F., 2013. The Role of Moral Values in Instigating Morally Responsible Decisions. J. Appl. Behav. Sci. 49, 69–94. https://doi.org/10.1177/0021886312471194

Comer, D.R., Vega, G., 2011. Moral Courage in Organizations: Doing the Right Thing at Work: Doing the Right Thing at Work. Routledge, New York. https://doi.org/10.4324/9781315702285

Peterson, C., Seligman, M.E.P., 2004. Character Strengths and Virtues. A Handbook and Classification. New York: Oxford University Press and Washingtom DC: American Psychological Association.

Sekerka, L.E., Bagozzi, R.P., 2007. Moral courage in the workplace: moving to and from the desire and decision to act. Bus. Ethics A Eur. Rev. 16, 132–149. https://doi.org/10.1111/j.1467-8608.2007.00484.x
